# Complete genome sequence of a new member of the genus *Badnavirus*, Dioscorea bacilliform RT virus 3, reveals the first evidence of recombination in yam badnaviruses

**DOI:** 10.1007/s00705-017-3605-9

**Published:** 2017-11-13

**Authors:** Moritz Bömer, Ajith I. Rathnayake, Paul Visendi, Gonçalo Silva, Susan E. Seal

**Affiliations:** Natural Resources Institute, University of Greenwich, Central Avenue, Chatham Maritime, Kent, ME4 4TB UK

**Keywords:** Yam, *Dioscorea* spp., *Dioscorea rotundata*, Episomal badnavirus, Endogenous *Dioscorea* bacilliform virus, Complete genome, Recombination, Phylogeny, West Africa

## Abstract

Yams (*Dioscorea* spp.) host a diverse range of badnaviruses (genus *Badnavirus*, family *Caulimoviridae*). The first complete genome sequence of Dioscorea bacilliform RT virus 3 (DBRTV3), which belongs to the monophyletic species group K5, is described. This virus is most closely related to Dioscorea bacilliform SN virus (DBSNV, group K4) based on a comparison of genome sequences. Recombination analysis identified a unique recombination event in DBRTV3, with DBSNV likely to be the major parent and Dioscorea bacilliform AL virus (DBALV) the minor parent, providing the first evidence for recombination in yam badnaviruses. This has important implications for yam breeding programmes globally.

**Electronic supplementary material:**

The online version of this article (doi:10.1007/s00705-017-3605-9) contains supplementary material, which is available to authorized users.

Yams (*Dioscorea* spp.) are an important staple food worldwide that play a major role in food security and income generation, particularly in West Africa [1]. Cultivated yam plants are propagated vegetatively through their tubers, resulting in the accumulation of viruses in yam germplasm and leading to an urgent need for a sustainable supply of virus-free planting material. The combination of vegetative propagation and the lack of strategic control measures promote the spread of viruses and the occurrence of multiple infections. The association of symptoms and the impact on yields attributable to individual viruses has as a result not been determined accurately to date.

*Dioscorea* bacilliform viruses (DBVs) (family *Caulimoviridae*, genus *Badnavirus*) are a concern for the safe movement of yam germplasm because of their high prevalence [2, 3]. Yam plants are hosts to a diverse range of badnaviruses, with recent findings suggesting the frequent occurrence of mixed infections in West African yam germplasm [4] as well as the presence of endogenous *Dioscorea* bacilliform viruses (eDBVs) as integrated forms of these viruses in *D. cayenensis-rotundata* genomes [5–7]. To date, seven distinct DBV genomes have been completely sequenced: Dioscorea bacilliform AL virus (DBALV), Dioscorea bacilliform AL virus 2 (DBALV2), Dioscorea bacilliform ES virus (DBESV), Dioscorea bacilliform RT virus 1 (DBRTV1), Dioscorea bacilliform RT virus 2 (DBRTV2), Dioscorea bacilliform TR virus (DBTRV), and Dioscorea bacilliform SN virus (DBSNV) [4, 8–11]. Several hundred partial badnavirus nucleotide sequences have also been generated by PCR using the badnavirus-specific primer pair Badna-FP/-RP [12], amplifying a 579 bp-fragment of the reverse transcriptase (RT)-ribonuclease H (RNaseH) domain used for taxonomic assessment of badnaviruses [13]. Phylogenetic analysis of these sequences led to the proposition of 15 badnavirus species whose members are associated with plants of the genus *Dioscorea* spp. According to the International Committee on Taxonomy of Viruses (ICTV), the demarcation criterion for species within the genus *Badnavirus* is sequence divergence of > 20% in a partial RT-RNaseH coding region [2–4, 6, 13].

A routine screening for episomal DBV infections of yam leaves showing viral symptoms was carried out by rolling circle amplification (RCA) in *D. rotundata* accessions maintained in the yam plant collection at the Natural Resources Institute (NRI, Chatham, UK), growing in conditions as described by Mumford and Seal [14]. For this, total nucleic acids were extracted from fresh yam leaf tissue using a modified CTAB method, as described by Kenyon et al. [2], and analysed by RCA following conditions described previously [4]. The yam breeding line TDr 89/02475 showed viral symptoms (mottling and chlorotic spots) associated with DBV and Yam mosaic virus (YMV) infections (Fig. 1A). This line was previously identified to be infected with DBRTV1 [4]. Restriction digestion of the RCA product of TDr 89/02475 using endonuclease BamH1 (NEB, UK) yielded the fragments of the expected sizes of 6.4 and 1.2 kbp for DBRTV1 (data not shown). To confirm the DBRTV1 infection in TDr 89/02475, we sequenced the partial RT-RNaseH domain used for classification of members of the genus *Badnavirus*. This was done by the excision and purification of the RCA fragments, followed by badnavirus-specific PCR using the Badna-FP/-RP primers and the RCA fragments as templates [4, 13]. The expected amplification product of 579 bp was obtained in a PCR using the 6.4-kbp fragment as template. Direct sequencing of the purified PCR product resulted in a mixture of sequences, and hence the PCR products were cloned into pGEM-T Easy Vector (Promega, UK). Five transformants were selected at random and sequenced. Three clones confirmed the expected DBRTV1 infection. However, the remaining two clones (A1-2 and A1-4) contained sequences that were 99% identical to NGl3841Dc (GenBank accession number KX008585), which was identified by RCA in our previous study [4]. The sequence NGl3841Dc was found to belong to the yam badnavirus monophyletic species group K5 defined by Kenyon et al. [2]. Sequencing of the complete episomal genome of this K5 yam badnavirus was undertaken. Outward-facing primers (DBRTV3-F/DBRTV3-R; see Fig. 1B and Table S1) were designed based on the partial RT-RNaseH sequences, and genomic TDr 89/02475 DNA was used as template for long PCR. The 50-µl PCR reaction mixture contained 1 µl of DNA template (~ 250 ng), 0.5 µM each primer, 0.25 mM each dNTP, 2.5 U of DreamTaq DNA polymerase and 1X DreamTaq Green buffer (Thermo Scientific, UK) containing 2 mM MgCl_2_. The cycle conditions for the long-PCR amplification were 95 °C for 5 min, followed by 30 cycles of 94 °C for 20 s, 58 °C for 30 s, 72 °C for 7 min, and a final extension of 72 °C for 7 min. These conditions generated a single PCR product of the expected 7-8 kbp size (data not shown), which was subsequently cloned using a TOPO^®^ XL Cloning Kit (Invitrogen, UK). The recombinant clone A9-6 was selected and fully sequenced twice using specific sequencing primers designed for genome walking (Table S1). A 7097-bp sequence was assembled using Geneious R10 (Biomatters, New Zealand). This sequence overlapped (115 bp at the 5’end, 55 bp at the 3’end) with the partial RT-RNaseH sequence present in clones A1-2 and A1-4. Combining these sequences resulted in a 7506-bp sequence (GenBank accession number MF476845) representing a consensus sequence of the entire viral genome of a new yam badnavirus member belonging to DBV species group K5.

**Fig. 1:**
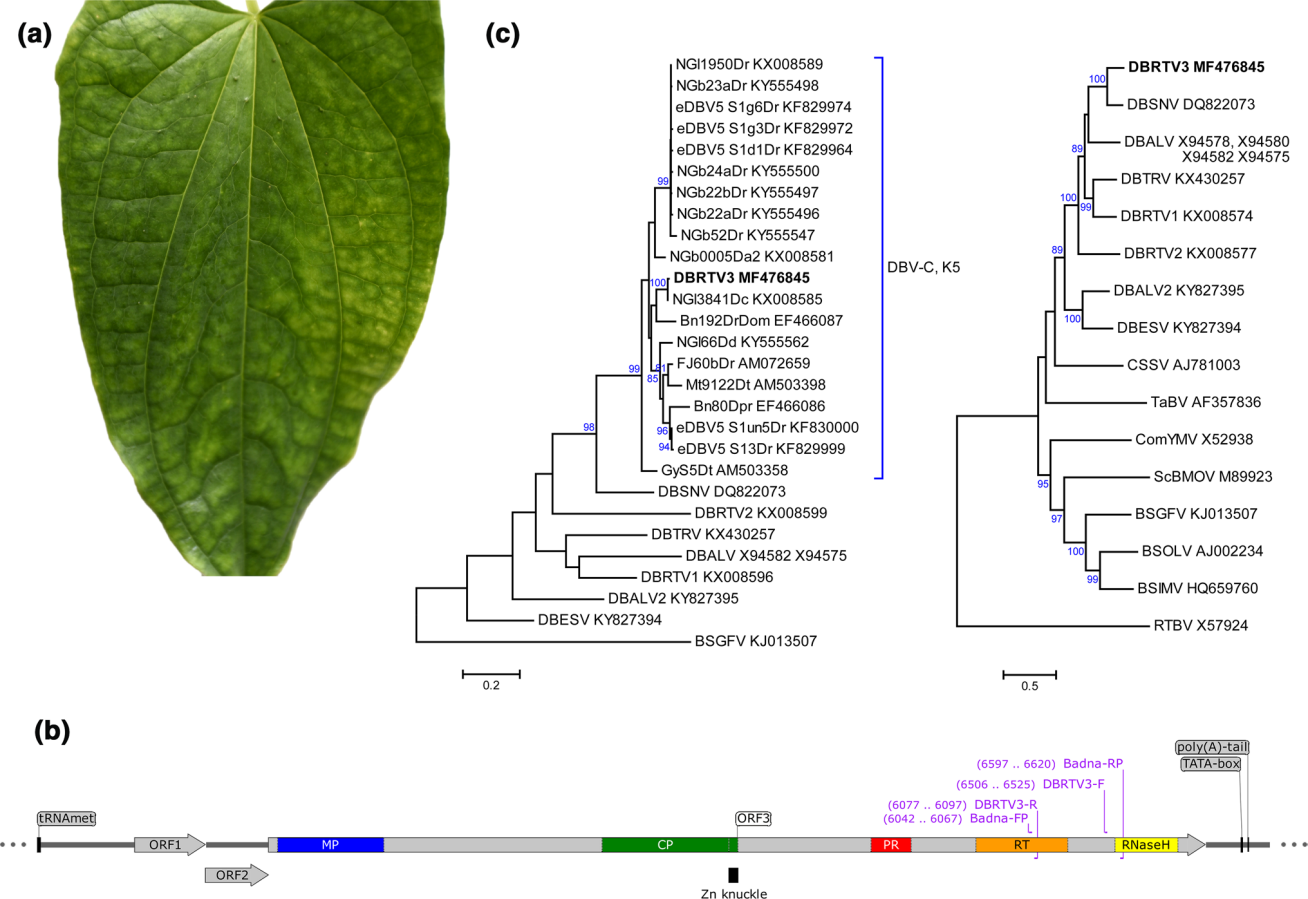
Characterization of Dioscorea bacilliform RT virus 3 (DBRTV3; GenBank accession number MF476845), detected in a *D. rotundata* plant. (**a**) Representative leaf showing viral symptoms of the diseased TDr 89/02475 yam breeding line from which the DBRTV3 virus genome was isolated. (**b**) Linear representation of the DBRTV3 genome organization showing binding sites of primers (purple) used in this study and the tRNA^Met^-binding site; the TATA-box; the putative poly(A) tail; open reading frame (ORF)1; ORF2; ORF3 with putative movement protein (MP), capsid protein zinc-finger domain (CP and Zn knuckle), pepsin-like aspartate protease (PR), reverse transcriptase (RT) and RNaseH conserved motifs. (**c**) Molecular phylogenetic analysis based on 528-bp-long partial nucleotide sequences of the badnavirus RT-RNaseH domain (left panel) of the DBV genomes and all 19 yam badnavirus sequences with nucleotide sequence identity values above 80% in similarity searches with the NCBI BLAST belonging to monophyletic species group K5 described by Kenyon et al. [2]. Banana streak GF virus (BSGFV) was used as an outgroup. The phylogenetic tree was constructed from full-length DBV genome sequences (right panel) and other badnavirus type members. Rice tungro bacilliform virus (RTBV) was used as an outgroup. GenBank accession numbers are provided, and DBRTV3 is highlighted in bold. Alignments were performed using Multiple Alignment using Fast Fourier Transform (MAFFT) [17], and the evolutionary relationships were inferred using the maximum-likelihood method based on the Hasegawa-Kishino-Yano model [22], conducted in MEGA7 [23]. Bootstrap values for 1000 replicates are given when above 80%. The scale bar shows the number of substitutions per base position

The consensus genome sequence (Fig. 1B) displayed all of the hallmarks of a typical badnavirus [13], and we propose the name “Dioscorea bacilliform RT virus 3” (DBRTV3) for this virus. DBRTV3 (7506 bp long) has a GC content of 43.3% and contains the expected putative host cytoplasmic initiator methionine tRNA (tRNA^Met^)-binding site (5’-TGGTATCAGAGCTTGGTT-3’) located within the intergenic region (IGR) at position 1-18 designating the beginning of the viral genome [15]. A potential TATA-box and a putative poly(A) tail were found within the IGR of DBRTV3 (Fig. 1B). Sequence analysis revealed three ORFs, where the start and stop codons of ORFs 1 and 2 and ORFs 2 and 3 overlapped by the ATGA motif in a -1 translational frame relative to the preceding ORF. No internal AUG codons were identified in ORF1 or 2, which agrees with the leaky scanning model of translation typical of members of the genus *Badnavirus* [13].

Analysis of deduced amino acid sequences identified proteins with molecular weights of 16.9, 14.3 and 215.7 kDa encoded by ORFs 1, 2 and 3, respectively. The ORF3 polyprotein of DBRTV3 has the characteristic features of members of the family *Caulimoviridae*, including the zinc knuckle (Zn knuckle), pepsin-like aspartate protease (PR), reverse transcriptase (RT), and ribonuclease H (RNaseH) (Fig. 1B) [13]. The coat protein (CP) and movement protein (MP) described by Xu et al. [16] were also located.

Molecular phylogenetic analysis based on 579-bp-long partial nucleotide sequences of the badnavirus RT-RNaseH domain of DBRTV3, DBALV, DBALV2, DBESV, DBRTV1, DBRTV2, DBTRV, DBSNV and all 19 yam badnavirus sequences available in the GenBank database with nucleotide identity values > 80% in similarity searches with the NCBI Basic Local Alignment Search Tool (BLAST) showed that DBRTV3 belongs to the monophyletic species group K5 described by Kenyon et al. [2] and is 99% identical to the sequence NGl3841Dc (Fig. 1C, left panel).

A phylogenetic tree was constructed from full-length DBV genome sequences and badnavirus type members of host plants other than yam (Fig. 1C, right panel). The resulting tree shows that (1) yam badnaviruses form a well-supported clade in which (2) DBALV2 and DBESV as well as DBTRV and DBRTV1 group closely together, as previously reported by Sukal et al. [9], and that (3) DBRTV3 and DBSNV represent sister taxa in the genus *Badnavirus*.

Sequence comparisons of DBRTV3 and all other fully sequenced episomal DBVs were performed (Table 1). The nucleotide sequence of the RT-RNaseH domain displayed 65.7% to 76.1% sequence identity to the corresponding region of the other DBV genomes, which is below the species demarcation criterion for the genus *Badnavirus* of 80% identity in this domain [13]. This confirms that the DBRTV3 sequence is the first complete genome sequence of a virus belonging to the previously described species group K5 [2]. Additional sequence comparisons confirmed that DBRTV3 is a distinct yam-infecting badnavirus, with the genome sequence of DBSNV (group K4) being the most similar.

**Table 1:** Percentages of nucleotide and amino acid sequence identity between DBRTV3 and DBALV, DBALV2, DBESV, DBRTV1, DBRTV2, DBSNV and DBTRV as described by Umber et al. [10]. Figures for amino acid sequences are shown in parentheses

	Complete genome	RT-RNaseH domain^a^	ORF1	ORF2	ORF3
DBALV	60.2%	66.3% (73.3%)	76.9% (84.7%)	55.4% (47.9%)	60.0% (61.3%)
DBALV2	51.0%	71.8% (72.2%)	53.1% (45.5%)	45.2% (35.9%)	55.5% (51.4%)
DBESV	48.7%	65.7% (68.8%)	53.4% (44.1%)	45.7% (32.8%)	51.8% (48.7%)
DBRTV1	59.2%	69.7% (75.6%)	66.9% (72.0%)	58.0% (53.2%)	63.0% (64.3%)
DBRTV2	55.4%	69.1% (70.5%)	61.2% (59.4%)	55.5% (50.4%)	59.0% (59.2%)
DBSNV	73.4%	76.1% (81.8%)	83.6% (91.0%)	72.7% (71.3%)	75.8% (82.2%)
DBTRV	60.4%	66.3% (75.0%)	71.3% (79.2%)	54.7% (54.9%)	61.8% (64.4%)

^a^ RT-RNaseH domain (528 bp long, excluding primer sequences and representing only complete amino acids) used for taxonomic assessment of badnaviruses [13] and typically amplified using the generic badnavirus primer pair Badna-FP/-RP [12]

Taking advantage of the growing number of complete yam badnavirus genome sequences falling into distinct DBV species groups, we performed recombination analysis using full-length DBV genome sequences, which were aligned using MAFFT [17] and then analysed in the RDP4 software package using default settings [18]. Of a total of 14 possible recombination events (Table S2), only a single event (Fig. 2) was detected with a very high degree of confidence by all seven recombination detection methods (RDP, GENECONV, BootScan, MaxChi, Chimaera, SiScan and 3Seq) available in RDP4 [18] all showing significant *p*-values (Table S2). The putative recombination site was located in the IGR of DBRTV3 and extended into the 5’end of ORF1. Significant differences in tree topologies were revealed by phylogenetic analysis of the recombined and non-recombined regions of the DBV genomes. A tree constructed using only the non-recombined region showed that DBRTV3 clustered together with DBSNV (Fig. 2, bottom panel), whereas DBRTV3 clustered with DBALV in a tree constructed using only the recombined region (Fig. 2, top panel). Therefore, DBRTV3 was identified to be the recombinant with DBSNV and DBALV as the viruses most closely related to the major and minor parent, respectively (Table S2). DBSNV was originally isolated from a wild *Dioscorea sansibarensis* plant in Benin [11], whereas DBALV was identified in a *D. alata* plant sampled in Nigeria [8]. The recombinant DBRTV3 originated from a *D. rotundata* breeding line maintained at the International Institute of Tropical Agriculture (IITA, Ibadan, Nigeria). Therefore, the opportunity for recombination between DBSNV and DBALV is not clear, but the literature suggests at least the latter is common throughout West Africa [3, 8].

**Fig. 2:**
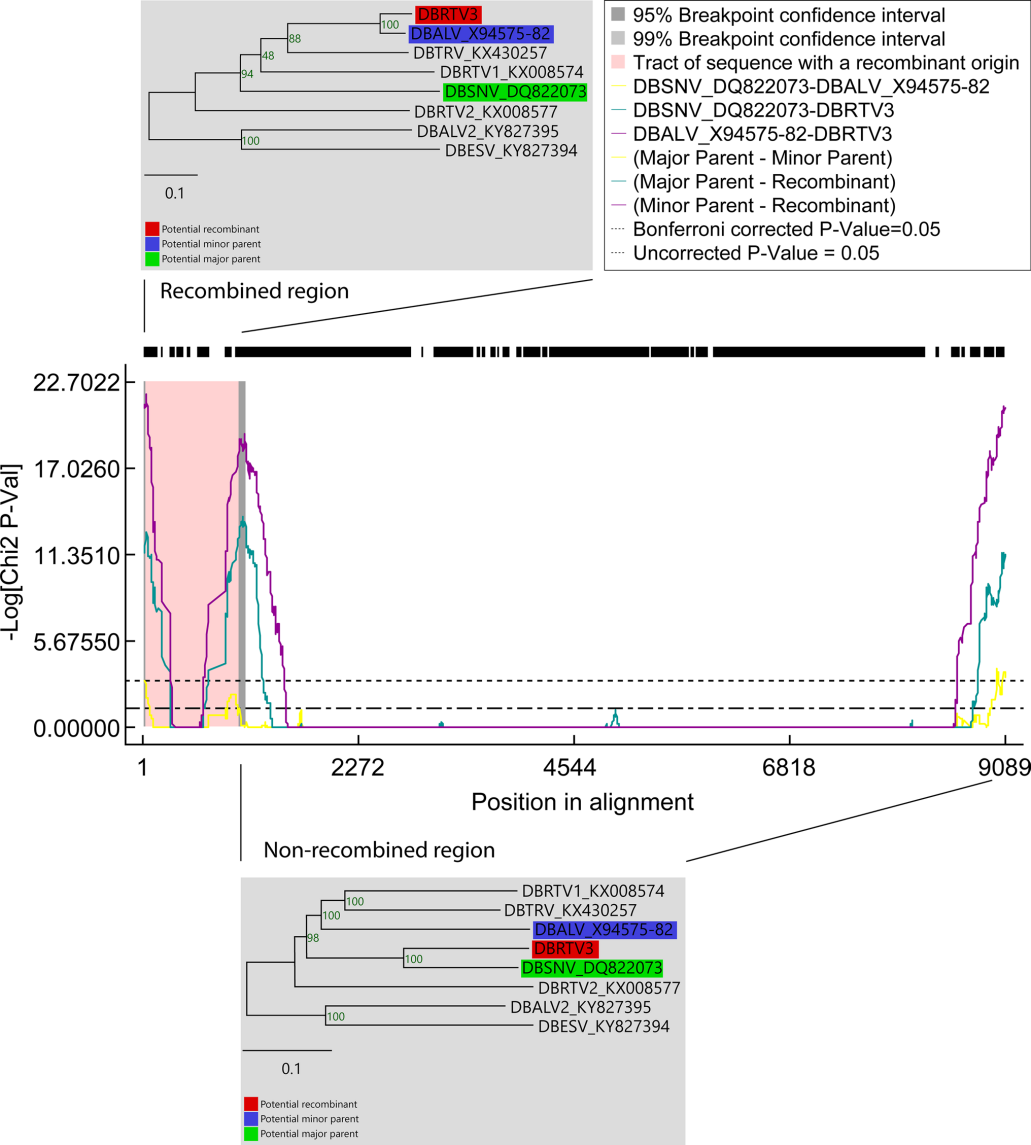
Recombination analysis of full-length DBV genome sequences using RDP4. A MaxChi plot shows a graphical overview of recombination event 1 in DBRTV3. DBRTV3 shows 77.2% sequence identity to DBSNV (major parent) in the non-recombined region of the genome but shares (89.8% identity) with DBALV (potential minor parent) only in the recombined region. Breakpoint positions mapping at positions 37 and 629 of the DBRTV3 genome sequence are indicated at the left and right boundaries of the shaded recombinant region. Neighbor-joining phylogenetic trees based on nucleotide sequences of the recombinant region (position 41-1014 in the sequence alignment) and the non-recombinant region (position 1-40 and 1015-9089 in the sequence alignment) show clustering of DBRTV3 with the potential minor parent DBALV (top panel) and with the major parent DBSNV (bottom panel), respectively

Recombination is an important driving force in viral evolution, and this study provides the first evidence for potentially extensive recombination in yam badnaviruses. It is interesting to note that four out of 14 possible recombination events were detected using parent-like sequences inferring unknown parents (Table S2), which suggests that the full genetic diversity of yam badnaviruses (complete genomes) is underestimated and unknown at present. The extent of recombination among DBV genomes will become clearer once more-extensive sequencing of episomal badnaviruses from West Africa and other yam-growing regions of the world has been performed.

Recombination in geminiviruses has previously been shown to originate from mixed infections [19]. Naturally occurring mixed infections of yam with more than one DBV isolate have been reported to be the norm recently [4, 10], and further studies of the phenomenon of recombination among DBVs can be expected to provide more detail about recombinant isolates in the future. Propagation of a wide assortment of yam germplasm at yam research centres and breeding programmes may facilitate recombination, as badnaviruses by themselves generally do not cause marked symptoms and hence may be cultivated in conditions that facilitate their transmission between germplasm, leading to recombination events and the emergence of more-virulent isolates. Therefore, there is an urgent need to develop reliable diagnostic tools for DBVs to help make rapid decisions on the health status of yam planting material, particularly in yam germplasm and seed yam distribution centres. For this, we plan to develop DBRTV3-specific diagnostic primers to be used in virus indexing assays. It remains remotely possible that DBRTV3 is an endogenous sequence that was inserted without rearrangement, a phenomenon that is occasionally found [20, 21]. Future work will be performed to test for the potential existence of eDBV forms of the DBRTV3 sequence in yam germplasm using Southern hybridization techniques similar to those described by Seal et al. [5] and Umber et al. [6].

In conclusion, the first complete genome sequence belonging to a member of yam badnavirus monophyletic species group K5 isolated from a *Dioscorea rotundata* breeding line is described. We propose this new member of the genus *Badnavirus* to be designated “Dioscorea bacilliform RT virus 3” (DBRTV3). Based on the comparison of full-length genome sequences, DBSNV was identified as the closest relative of DBRTV3. DBSNV was also found to be the major parent in a unique recombination event identified in DBRTV3, with DBALV likely to be the minor parent. The results provide the first evidence for recombination among yam badnavirus genomes. This finding implies that breeding programmes should introduce strict control measures to prevent the transmission of badnaviruses from one yam breeding line to avoid the potential creation of mixed infections that could lead to recombinant badnaviruses with increased virulence.

## Electronic supplementary material

Below is the link to the electronic supplementary material.

Supplementary material 1 (TXT 7 kb)

Supplementary material 2 (XLSX 9 kb)

Supplementary material 3 (XLSX 11 kb)
